# Type 3 inositol 1,4,5-trisphosphate receptor has antiapoptotic and proliferative role in cancer cells

**DOI:** 10.1038/s41419-019-1433-4

**Published:** 2019-02-22

**Authors:** Ingeborg Rezuchova, Sona Hudecova, Andrea Soltysova, Miroslava Matuskova, Erika Durinikova, Barbora Chovancova, Michal Zuzcak, Marina Cihova, Monika Burikova, Adela Penesova, Lubomira Lencesova, Jan Breza, Olga Krizanova

**Affiliations:** 1 Institute of Virology, Biomedical Research Center, SAS, Bratislava, Slovakia; 2 Institute of Clinical and Translational Research, Biomedical Research Center, SAS, Bratislava, Slovakia; 3 Faculty of Natural Sciences, Comenius University, Bratislava, Slovakia; 4 Cancer Research Institute, Biomedical Research Center, SAS, Bratislava, Slovakia; 5 Department of Urology with Kidney Transplant Center, Faculty of Medicine, University Hospital, Bratislava, Slovakia; 6 Department of Chemistry, Faculty of Natural Sciences, University of Ss. Cyril and Methodius, Trnava, Slovakia

## Abstract

Although the involvement of type 1 (IP_3_R1) and type 2 (IP_3_R2) inositol 1,4,5-trisphosphate receptors in apoptosis induction has been well documented in different cancer cells and tissues, the function of type 3 IP_3_R (IP_3_R3) is still elusive. Therefore, in this work we focused on the role of IP_3_R3 in tumor cells in vitro and in vivo. We determined increased expression of this receptor in clear cell renal cell carcinoma compared to matched unaffected part of the kidney from the same patient. Thus, we hypothesized about different functions of IP_3_R3 compared to IP_3_R1 and IP_3_R2 in tumor cells. Silencing of IP_3_R1 prevented apoptosis induction in colorectal cancer DLD1 cells, ovarian cancer A2780 cells, and clear cell renal cell carcinoma RCC4 cells, compared to apoptosis in cells treated with scrambled siRNA. As expected, silencing of IP_3_R3 and subsequent apoptosis induction resulted in increased levels of apoptosis in all these cells. Further, we prepared a DLD1/IP_3_R3_del cell line using CRISPR/Cas9 gene editing method. These cells were injected into nude mice and tumor's volume was compared with tumors induced by DLD1 cells. Lower volume of tumors originated from DLD1/IP_3_R3_del cells was observed after 12 days, compared to wild type DLD1 cells. Also, the migration of these cells was lesser compared to wild type DLD1 cells. Apoptosis under hypoxic conditions was more pronounced in DLD1/IP_3_R3_del cells than in DLD1 cells. These results clearly show that IP_3_R3 has proliferative and anti-apoptotic effect in tumor cells, on contrary to the pro-apoptotic effect of IP_3_R1.

## Introduction

Intracellular calcium ions act as a second messenger to regulate gene transcription, cell proliferation, migration, and cell death. Targeting detailed calcium signaling for cancer therapy has become an emerging research area.

Inositol 1,4,5-trisphosphate (IP_3_) receptors (IP_3_Rs) are intracellular calcium channels that are able to release calcium from intracellular stores upon activation by IP_3_ and modulation by calcium. Three different IP_3_R isoforms are expressed in different amounts in various cells, and different isoforms are capable of forming homo- and heterotetramers^1^. IP_3_Rs are emerging as key sites for the regulation of pro- and anti-apoptotic factors^2^. In addition to the direct role of IP_3_Rs in the initiation of apoptosis by providing a conduit for endoplasmic reticulum to mitochondria calcium transfer, there are several additional feedback mechanisms that have been proposed and allow IP_3_Rs to play a role in amplifying calcium-dependent apoptotic pathways^3^. Until now, the involvement of IP_3_Rs in the process of apoptosis has been mainly assigned to IP_3_R1^4–6^ and IP_3_R2^7,8^. Nevertheless, the function of the type 3 IP_3_Rs (IP_3_R3) is still elusive; both pro-apoptotic and anti-apoptotic effects were ascribed to this type of receptor^9–14^. Up to now, the expression of the IP_3_R3 subtype was shown to correlate with colorectal carcinoma aggressiveness^9^, or with increased cell migration capacities^12^. Inhibition of the IP_3_R3 subtype reduced breast cancer cell proliferation^10^, migration, invasion, and survival of glioblastoma cells^11^ and revealed an oscillating Ca^2+^ signature along with a slowing down cell migration in human breast cancer cells^12^. IP_3_R3 may also be specifically involved in gastric cancer peritoneal dissemination and these receptors may serve as a molecular target for treatment of this cancer^13^. On the other hand, inhibition of the IP_3_R3 degradation resulted in sensitization to photodynamic therapy in tumors with no or low levels of phosphatase and tensin homologue (PTEN) expression^14^.

All above-mentioned results strongly point to differences among the function of IP_3_R1 (which is known to participate in inner-mitochondrial-pathway of apoptosis) and IP_3_R3. Therefore, we aimed to study the relevance of IP_3_R3 in tumors. We compared the expression of individual IP_3_R’s type in clear cell renal cell carcinoma (ccRCC) tumors. Further, we studied the effect of silencing of individual types of IP_3_Rs on apoptosis in stable cell lines derived from colorectal carcinoma (DLD1), ovarian cancer (A2780) and ccRCC (RCC4) in vitro. Finally, we compared tumorigenicity of DLD1 and DLD1/IP_3_R3_del cells using subcutaneous xenograft model.

## Materials and methods

### Patients

In total, 23 primary tumor samples and normal adjacent synonym tissue were collected from patients diagnosed with ccRCC. Patients were treated at the Department of Urology with Kidney Transplant Center Faculty of Medicine, Comenius University Bratislava and University Hospital Bratislava. The study was approved by the Ethics Committee of the Biomedical Research Center SAS nr. EK/BmV-01/2016 and University Hospital Bratislava, Slovakia, nr. EK 131/17, in agreement with the Ethical guidelines of the Declaration of Helsinki as revised in 2000.

All patients underwent radical nephrectomy, finally in 18 patients (12 males/6 females, average age 62.4 ± 3.1 years), the ccRCC was histopathologically confirmed. Fuhrman grades were as follows: grade I in 2 samples, grade II in 8 samples, grade III in 1 sample, and grade IV in 3 samples, tumor grade of the rest of the patients was unknown. Just two patients were suffering from metastases—one of grade 3 (T3bN2M1) and one of grade 4 (T4N0M1). Morphology of the rest of the kidney was normal in all patients, as determined by the pathologist. After nephrectomy, tumor mass and also corresponding healthy part of tissue was immediately taken into the RNA Latter® and kept at 4 °C until isolation. Tumor sample (ca. 0.5 cm^2^) was cut off from the outer part of the tumor and corresponding unaffected tissue was taken from the distinct part of the extirpated kidney.

### Microarray assays

100 ng of total RNA was transcribed into cDNA. Subsequently labeling reaction using Quick Amp Labeling kit, where Cy3-dCTP (unaffected tissue samples) and Cy5-dCTP (tumor samples) were used to obtained cRNA. For hybridization, SurePrint G3 Human Gene Expression 8 × 60 K v2 Microarray Slide were used (Agilent Technologies, USA). Further, microarray was performed as described in Soltysova et al.^15^.

### Cell cultivation

For experiments, human colon adenocarcinoma cell line DLD1 (ATCC, CCL-221), human renal cell carcinoma cell line RCC4 (ECACC, 03112702), and/or human ovarian cancer cell line A2780 (Sigma-Aldrich, 93112519) were cultured in Minimal Essential Medium of Dulbecco (DMEM; Sigma, USA) or RPMI medium (Sigma, USA) with a high glucose (4.5 g L^−1^) and L-glutamine (300 μg mL^−1^), supplemented with 10% fetal bovine serum (Sigma, USA), penicillin (Calbiochem, USA; 100 U mL^−1^), and streptomycin (Calbiochem, USA; 100 μg mL^−1^). Cells were cultured in a water-saturated atmosphere at 37 °C and 5% CO_2_. In some groups, apoptosis was induced by apoptosis inducer set I (AIK). Apoptosis Inducer Set I (AIK; Calbiochem, cat. nr. 178486), is composed of 5 ready-to-use chemical reagents that induce apoptosis through different mechanisms: Actinomycin D (inhibits RNA synthesis), Camptothecin (an inhibitor of nuclear topoisomerase), Cycloheximide (an inhibitor of protein synthesis), Dexamethasone (induces apoptosis probably by binding and activating the intracellular glucocorticoid receptor), and Etoposide (inhibits topoisomerase activity). Apoptosis is known to be induced either by inner, mitochondrial pathway (where IP_3_R1 plays a crucial role), or by outer apoptotic pathways through “death receptors”. The AIK treatment results in activation of inner, mitochondrial pathway and expression of the IP_3_R1 is upregulated^16^.

### Gene silencing

Cells were grown in 6-well plates in RPMI with 10% FBS. Transfection of siRNAs was performed with DharmaFECT1 transfection reagent (Dharmacon, Thermo Scientific, USA) as described previously in Hudecova et al.^6^. ON-TARGET plus SMART pool human ITPR1 and ITPR3 siRNAs (Dharmacon, Thermo Scientific, USA) were applied to the final concentration of 100 pmol per well. The same procedure was performed with ON-TARGET plus Non-targeting Pool, which serves for the determination of baseline cellular responses in RNAi experiments. Based on the previous calibration, silencing was performed for 48 h. After the first 24 h of silencing, apoptosis inducer kit (AIK) was applied for an additional 24 h. Finally, all groups of cells were harvested and used in further experiments.

The efficiency of the IP_3_R1 and IP_3_R3 silencing was measured by Western blot analysis, as described below.

### Detection of apoptosis with Annexin-V-FLUOS

Cells were gently scraped and pelleted at 1000 × *g* for 5 min and then washed with 1 mL of phosphate saline buffer (PBS; pH 7.4). Cell pellet was resuspended in 200 μL of Annexin-V-FLUOS/propidium iodide labeling solution (Roche Diagnostics, USA) and incubated at room temperature in dark for 20 min according to the manufacturer’s protocol. After the incubation, samples were placed on ice and measured on BD FACSCanto II flow cytometer (Becton Dickinson, Ann Arbor, USA). Results were evaluated with a Flowing software version 2.5.1.

### Generation of IP_3_R3-knockout and IP_3_R1/IP_3_R3-double-knockout DLD1 cell lines

IP_3_R3-knockout DLD1 cell line, hereafter called DLD1/IP_3_R3_del, was established by using the CRISPR/Cas9 (CRISPR (clustered, regularly interspaced, short palindromic repeats)/Cas9 (CRISPR-associated protein 9)) gene editing method. The IP_3_R3 CRISPR guide RNA sequences (GTGCCCCATGAACCGCTACT and TACGAGCTCAGCGACAACGC) were designed by the laboratory of Feng Zhang at the Broad Institute in order to efficiently target the IP_3_R3 gene with minimal risk of off-target Cas9 binding elsewhere in the genome^17,18^. Lentiviral transfer plasmids pLCS-ITPR3-1 and pLCS-ITPR3-2 (GenScript, USA) contained a lentiCRISPRv2 backbone and single above-mentioned oligos cloned into the single guide RNA (sgRNA) scaffold. To make lentiviruses, transfer plasmids pLCS-ITPR3-1 or pLCS-ITPR3-2 were co-transfected into HEK293T cells with the packaging plasmids pVSVg (Addgene) and psPAX2 (Addgene). Virus-containing medium was collected after 48, 60, and 72 h and passed through a 0.45 μm low protein-binding filter. Lentiviruses were concentrated using PEG 6000 and sedimented by centrifugation (1500 × *g*, 4 °C for 30 min). DLD1 cells, plated the day before at a density of 0.25 × 10^6^ cells per 6 cm plate, were infected with each lentivirus or their combination. Twenty-four hours after transduction, cells were selected in puromycin (Puromycin, InvivoGen) and then the IP_3_R3 protein knockout was confirmed by immunofluorescence (IF), western blotting (WB), and sequencing.

IP_3_R31/IP_3_R3-double-knockout DLD1 cell line, hereafter called DLD1/IP_3_R1/IP_3_R3_del, was generated by infection of DLD1/IP_3_R3_del cells with Edit-R Human ITPR1 Set of 3 Lentiviral sgRNA (Dharmacon) lentivirus particules at low multiplicity of infection = 0.3 CFU/cell. Individual cell clones were generated by serial dilution cloning method with puromycin (Puromycin, InvivoGen) selective pressure in a 96-well plate. The IP_3_R1/IP_3_R3 double protein knockout in expanded single cell clones was confirmed by western blot analysis (WB).

### Immunofluorescence

DLD1 and DLD1/IP_3_R3_del cells grown on glass coverslips (amount—1.3 × 10^4^) were fixed in ice-cold methanol. Immunofluorescence was performed as described in Hudecova et al.^6^. with rabbit polyclonal IP_3_R3 (ab55983, Abcam, Cambridge, UK) primary antibody diluted 1:50 and 1:100 in PBS with 1% bovine serum albumin.

### Western blot analysis

Cells were scraped and resuspended in 10 mM Tris–HCl pH 7.5, 1 mM PMSF and subjected to centrifugation for 10 min at 10,000 × *g* and 4 °C. The pellet was resuspended in Tris buffer containing the 50 µM CHAPS detergent, and incubated for 20 min at 4 °C. The lysate was centrifuged for 10 min at 10,000 × *g* at 4 °C. Protein concentration in supernatants was determined by the method of Lowry^19^. Protein extract from each sample was separated by electrophoresis on gradient SDS polyacrylamide gels and proteins were transferred to Hybond-P membrane using semidry blotting (Owl, Inc.). The membranes were blocked in 5% non-fat dry milk in TBS-T overnight at 4 °C and then incubated with primary antibodies raised against the following proteins: rabbit polyclonal IP_3_R1 (I157, Sigma-Aldrich, USA), rabbit polyclonal IP_3_R3 (ab55983, Abcam, Cambridge, UK), and mouse monoclonal beta-actin (ab6276, Abcam, Cambridge, UK). Horseradish peroxidase-linked secondary antibody and chemiluminescence detection system (LuminataTM Crescendo Western HRP Substrate, Millipore) was used for visualization. Each membrane was digitally captured using an imaging system (C-DiGit, LI-COR).

### Proximity ligation assay

The proximity ligation assay (PLA) was used for in situ detection of the interaction between IP_3_R1/IP_3_R3. The assay was performed in a humid chamber at 37 °C according to the manufacturer’s instructions (Olink Bioscience, Sweden). DLD1 and DLD1/IP_3_R3_del cells were seeded on glass coverslips. Afterwards, the cells were fixed with methanol, blocked with 3% BSA/PBS for 30 min, incubated with a mixture of antibodies against IP_3_R1 and IP_3_R3 for 1 h, washed three times, and incubated with plus and minus PLA probes for 1 h. Then, the cells were washed (3 × 5 min), incubated for 40 min with ligation mixture containing connector oligonucleotides, washed again, and incubated with amplification mixture containing fluorescently labeled DNA probe for 100 min. After a final wash, the samples were mounted and the signal representing the interaction between IP_3_R1 and IP_3_R3 was determined by confocal microscope imaging system TCS SPE-II (Leica, Wetzlar, Germany) with 405 and 532-nm lasers for excitation. To measure the intensity of fluorescence, software—LAS AF (Leica Application Software platform for confocal microscope) was used. Mouse monoclonal antibody IP_3_R1 (407140, Calbiochem, USA) and rabbit polyclonal IP_3_R3 (HPA003915, Sigma-Aldrich, USA) were used in the experiment.

### Chemically induced hypoxia

Hypoxia was induced by 100 µM dimethyloxalylglycine (DMOG; Cayman Chemical Company, USA) for 24 and 48 h. DMOG is a cell permeable competitive inhibitor of HIF-alpha prolyl hydroxylase (HIF-PH) leading to the stabilization of HIF and subsequent angiogenesis and glucose metabolism at concentrations between 0.1 and 1 mM.

### Cytosolic [Ca2+]i staining by FURA2 AM fluorescent dye

Cells were plated on a 24-well plate at the density of 4 × 10^4^. After treatment, the cells were washed with 1 mL of serum-free medium and loaded with 20 µM FURA2 AM; (Sigma-Aldrich, USA) in the presence of 0.5% pluronate (Sigma-Aldrich, USA) and 0.1 nM ionomycine in serum-free medium for 40 min at 37 °C in the dark. The cells were then washed three times with a 500 µL of PBS. Fluorescence was measured on the fluorescence scanner Synergy II (BioTek, Germany) at λex 340/380 nm and λem 516 nm. The results were calculated as the ratio between 340 and 380 nm and expressed as relative fluorescence units (RFU).

### TUNEL assay

Tissue cryosections (5 µm in thickness) were prepared from the tumor samples from nude mice, and stained using Fluorescein in situ cell death detection kit (Roche, Germany) according to the standard protocol provided by the manufacturer (Roche 11684795910, version 17). Nuclei were counterstained using DAPI solution (20 µg/mL, Sigma-Aldrich, USA). Staining patterns were analyzed with Zeiss fluorescent microscope and automated imaging Metafer (MetaSystems GmbH, Germany) (magnification, ×20). Tissue section was stained with hematoxylin and eosin (HaE) (Diapath, Italy). Digital images were captured and analyzed with Leica DM 5500. Images were acquired using a Leica DFC 340 FX camera (magnification, ×25).

### Scratch assay

One hundred thousand DLD1 cells and 65,000 DLD/IP_3_R3_del cells per well were plated in octaplicates on ImageLock 96-well plates (Essen BioScience, UK), and let to adhere for 24 h. Confluent monolayers were then wounded with wound making tool (IncuCyte WoundMaker; Essen BioScience), washed twice and supplemented with fresh culture medium. Images were taken every 2 h for the next 48 h in the IncuCyte ZOOM™ kinetic imaging system (Essen BioScience). Cell migration was evaluated by IncuCyte ZOOM™ 2016A software based on the relative wound density measurements and expressed as means of octaplicates ± SEM.

### In vivo experiments

Animal experiments were approved by the Institutional Ethic Committee and by the national competence authority—State Veterinary and Food Administration of the Slovak Republic (Project Registration No. Ro 1289/18-221) in compliance with the Directive 2010/63/EU and the Regulation 377/2012 on the protection of animals used for scientific purposes. Project was conducted in the approved animal facility (License No. SK UCH 02017). Athymic (Balb/c nu/nu) mice were bilaterally s.c. injected by 5 × 10^6^ DLD-1 or DLD1/IP_3_R3_del cells resuspended in 100 µL PBS. Animals were regularly inspected for tumor incidence. Growing xenografts were measured by the caliper, and tumor volume was calculated according to the formula volume = (length × width^2^)/2. Results were evaluated as mean volume ± SD. At the experimental endpoint (12 days after injection) mice were sacrificed and xenografts were used for Western blot analysis.

### Statistical analysis

The results are presented as mean ± SEM. Each value represents an average of at least 3 wells from at least three independent cultivations of each type of cells. Statistical differences among groups were determined by ANOVA. Statistical significance * or +—*p* < 0.05 was considered to be significant, ** or ^++^*p* < 0.01, *** or ^+++^*p* < 0.001. For multiple comparisons, an adjusted *t*-test with *p* values corrected by the Bonferroni method was used (InStat, GraphPad Software).

## Results

**Fig. 1:**
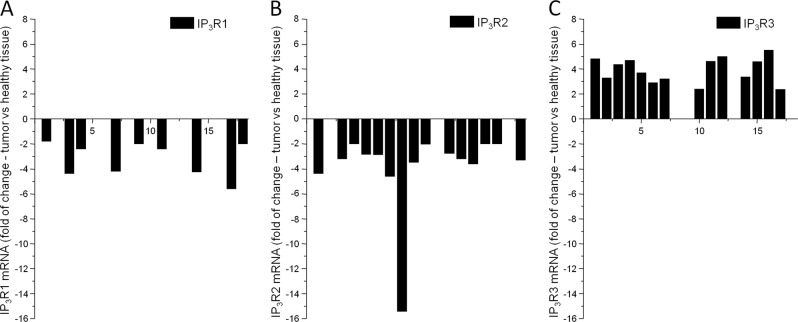
Changes of the expression of IP_3_ receptors in ccRCC vs normal kidney tissue. Changes in mRNA levels of the IP_3_R1 (**a**), IP_3_R2 (**b**), and IP_3_R3 (**c**) in ccRCC tumors of 18 patients compared to mRNA levels from the unaffected part of their kidney. From 18 patients, IP_3_R1 mRNA in ccRCC was decreased in 9 patients and was not affected in 9 patients compared to their unaffected part of the kidney (**a**). Levels of IP_3_R2 mRNA in ccRCC were decreased in 15 patients and not changed in 3 patients (**b**). The IP_3_R3 mRNA was increased in ccRCC tumors of 14 patients and not changed in 4 patients (**c**)

In a group of 18 patients, we evaluated the expression of the IP_3_R1, IP_3_R2, and IP_3_R3 in ccRCC tumors compared to the unaffected part of tissue from the same patient (Fig. 1) using microarray technique. In these tumors, we observed decreased or unchanged expression of the IP_3_R1 in tumors compared to unaffected part of kidney (Fig. 1a), while gene expression of the IP_3_R2 was mostly downregulated (Fig. 1b) and the expression of the IP_3_R3 was predominantly upregulated (Fig. 1c) in ccRCC compared to corresponding unaffected tissue.

**Fig. 2:**
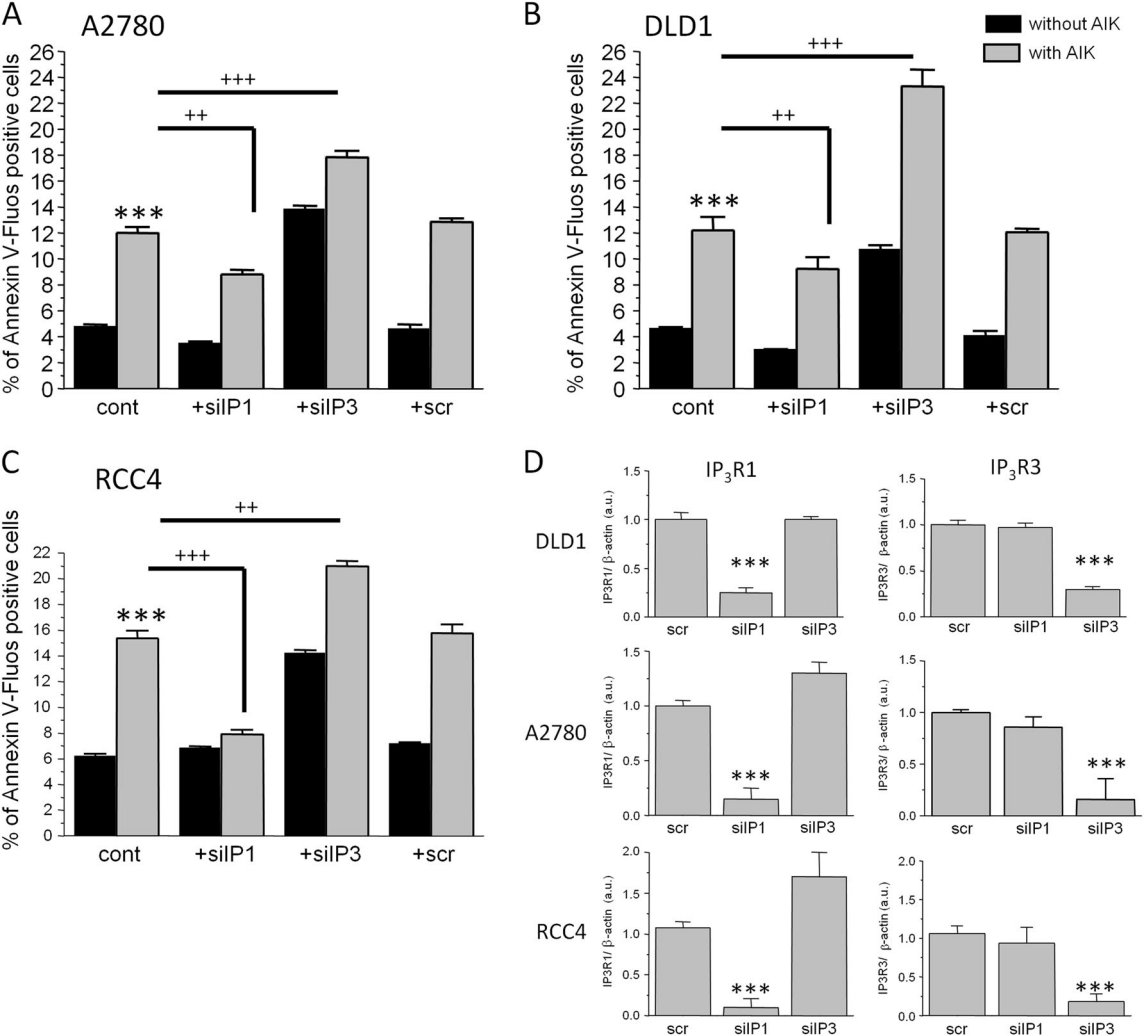
Determination of apoptosis after silencing of type 1 and 3 IP_3_ receptors. Detection of apoptosis after silencing of the IP_3_R1 (silIP1) and/or IP_3_R3 (silIP3) in ovarian cancer A2780 cells (**a**), colorectal carcinoma DLD1 cells (**b**), and clear cell renal cell carcinoma RCC4 cells (**c**) and after apoptosis induction (AIK) in the cells with silenced IP_3_R1 (silIP1) and/or IP_3_R3 (silIP3). Silencing and subsequent AIK induces a significant increase in apoptosis, compared to unaffected A2780, DLD1, and RCC4 cells (cont). In cells with silenced IP_3_R1 and subsequently treated for 24 h with AIK, apoptosis was significantly lower than in non-silenced cells treated with AIK. Opposite, when IP_3_R3 was silenced, apoptosis was higher compared to non-silenced A2780, DLD1, and also in RCC4 cells. As a control of potential siRNA toxicity, scrambled non-coding primer (scr) was used. Effectivity of silencing of the IP_3_R1 as determined by Western blot analysis was in DLD1 cells approximately 75%, in A2780 cells approximately 85%, and in RCC4 approximately 85%. Effectivity of silencing of the IP_3_R3 in DLD1 cells was approximately 65%, in A2780 approximately 70%, and in RCC4 approximately 80% (**d**). Results are displayed as mean ± SEM, *n* = 3–6. Statistical significance compared to control cells ***p < 0.0001. Statistical significance compared to AIK treated cells ^++^*p* < 0.001 and ^+++^*p* < 0.0001

In order to verify different effect of the IP_3_R1 and IP_3_R3 on apoptosis induction, we silenced either IP_3_R1, or IP_3_R3 separately and we also compared the level of apoptosis in A2780 (Fig. 2a), DLD1 (Fig. 2b), and RCC4 (Fig. 2c) cells after apoptosis induction by a mixture of apoptotic inducers (AIK; Fig. 2). AIK increased significantly apoptosis in all three types of cells. Silencing of the IP_3_R1 in all above-mentioned cells has no effect on apoptosis compared to control cells, but silencing of the IP_3_R3 resulted in upregulation of apoptosis in RCC4, A2780, and DLD1 cells. Silencing of the IP_3_R1 and subsequent treatment with AIK resulted in lower level of apoptosis compared to groups treated with only AIK. As expected, silencing of the IP_3_R3 and subsequent treatment with AIK caused increased levels of apoptosis compared to groups treated with only AIK (Fig. 2a–c). Effectivity of the IP_3_R1 silencing was approximately 75–85% and IP_3_R3 silencing was approximately 65–80%, depending on the type of cells (Fig. 2d), as determined by Western blot analysis.

**Fig. 3:**
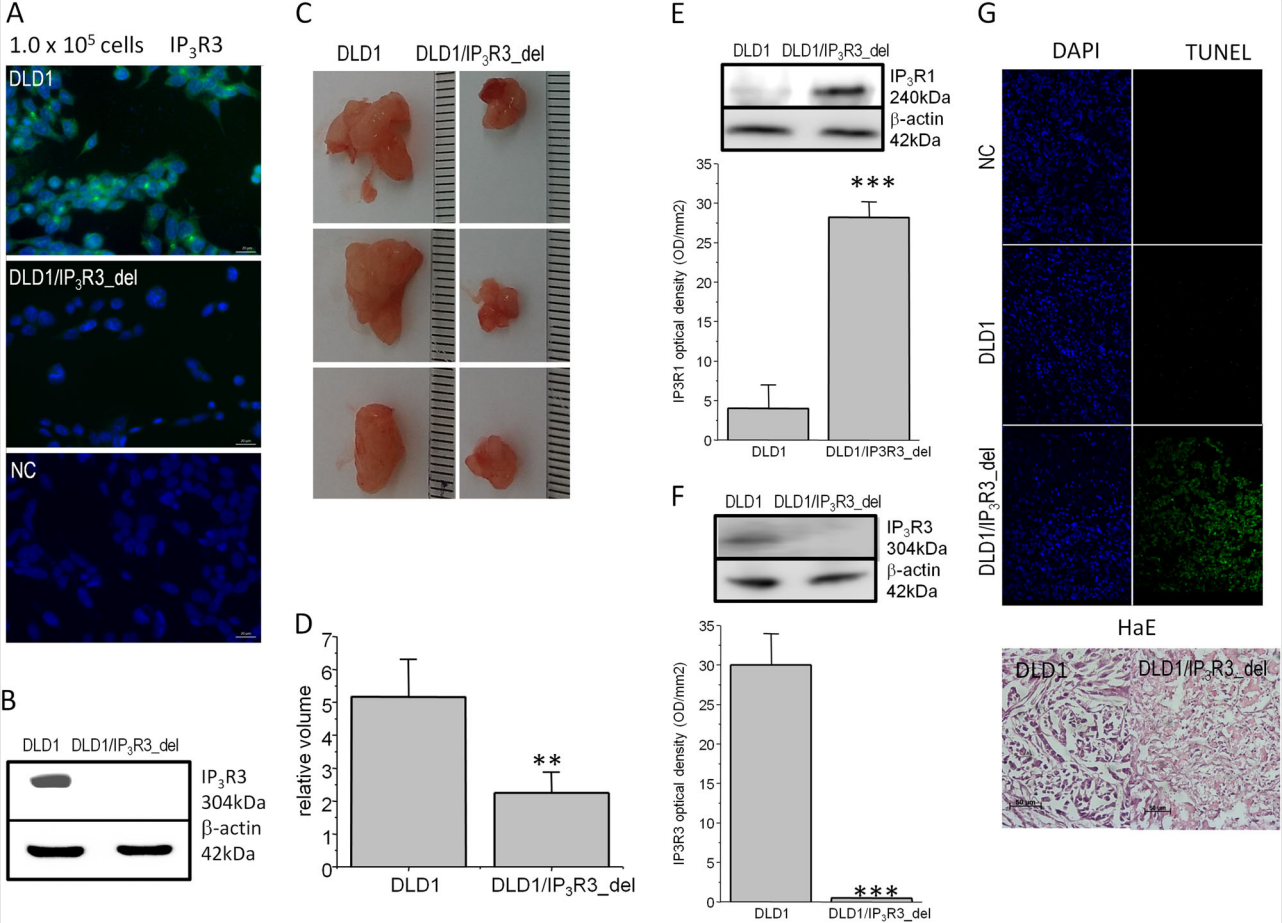
Tumor’s induction in athymic nude mice by DLD1 cells with/without IP_3_R3. IP_3_R3-knockout DLD1 cell line, DLD1/IP_3_R3_del, was established using the CRISPR/Cas9 gene editing method. The IP_3_R3 protein knockout in DLD1/IP_3_R3_del cells was confirmed by immunofluorescence (**a**) and also by Western blot analysis (**b**) using anti-IP_3_R3 antibody. Either DLD1, or DLD1/IP_3_R3_del cells were subcutaneously injected into the lower flank of the nude mice and growth of tumors was compared (**c**). After 12 days, tumors were extirpated (**c**) and relative volumes were significantly lower from DLD1/IP_3_R3_del cells compared to DLD1 cells (**d**). Western blot analysis revealed increased expression of the IP_3_R1 in tumors from DLD1/IP_3_R3_del cells compared to DLD1 cells (**e**), while no expression of the IP_3_R3 was observed in tumors induced by DLD1/IP_3_R3_del cells (**f**). Apoptosis was determined in tumor slices by TUNEL assay (**g**), and differenced in morphology are shown by hematoxylin/eosin staining (HaE; **g**, bottom). NC negative control. In graphs, results are displayed as mean ± SEM, *n* = 6. Statistical significance *p < 0.05, **p < 0.001, and ***p < 0.0001

To evaluate the physiological relevance of the IP_3_R3, we decided to knockout the IP_3_R3 and compare tumor induction of knockout IP_3_R3 cells with normal cells. Unfortunately, since RCC4 cells have a very low tumorigenicity^20^, and also our experience, we constructed DLD1 cells with depleted IP_3_R3 by CRISPR/Cas9 gene editing method (DLD1/IP_3_R3_del). Effectivity of CRISPR/Cas9 depletion of IP_3_R3 was determined by immunofluorescence using a corresponding primary antibody against IP_3_R3 (Fig. 3a) and also by WB analysis (Fig. 3b). Athymic nude mice were subcutaneously inoculated either by a wild type (DLD1) cells or cells with depleted IP_3_R3 (DLD1/IP_3_R3_del). After 12 days, size of the tumors was assessed (Fig. 3c). We observed a significant decrease of tumor volume, when mice were inoculated with DLD1/IP_3_R3_del cells, compared to DLD1 cells (Fig. 3d). Xenografts were used for determination IP_3_R1 and IP_3_R3 proteins (Fig. 3e, f). We observed increased expression of the IP_3_R1 in xenografts from DLD1/IP_3_R3_del cells, compared to DLD1 cells (Fig. 3e) and no expression of the IP_3_R3 in xenografts from DLD1/IP_3_R3_del cells (Fig. 3f). Also, apoptosis determined in tumor's slices by TUNEL assay was visible in DLD1/IP_3_R3_del cells, but not in DLD1 cells (Fig. 3g). In the HaE staining assay, nuclei and their fragments of tumor cells are violet and the cytoplasm is pink-red (Fig. 3g, bottom). Tumor tissues from mice treated with DLD1 cells displayed typical tumor tissue pattern with violet color, and the tumor tissues in DLD1/IP_3_R3_del cell's xenograft changed clearly from violet to pink-red. These results demonstrated that the therapeutic effect of potential IP_3_R3 inhibitor is due to the induction of tumor cells apoptosis.

**Fig. 4:**
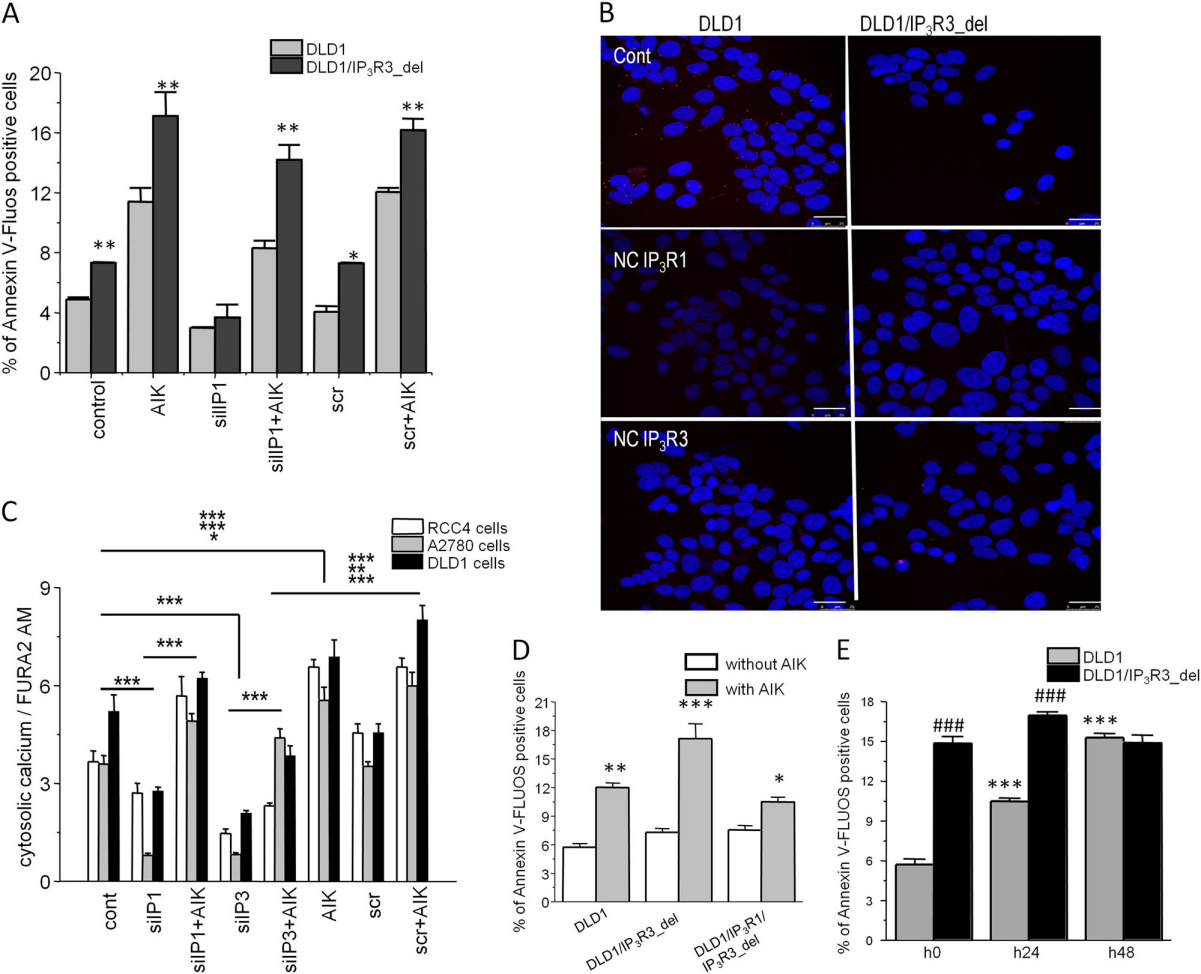
Determination of apoptosis, cytosolic calcium and co-localization of IP_3_R1 and IP_3_R3. Apoptosis induction in DLD1 and DLD1/IP_3_R3_del cells after silencing of the IP_3_R1 and subsequent induction of apoptosis by AIK (**a**). Silencing of the IP_3_R1 decreased the basal apoptosis compared to cells treated with scrRNA in both, DLD1 and DLD1/IP_3_R3_del cells. After treatment with AIK, apoptosis was significantly higher in DLD1/IP_3_R3_del than in DLD1 cells. In DLD1 cells, we observed co-localization of IP_3_R1 and IP_3_R3 by proximity ligation assay (**b**). Scale bar represents 25 μm. Silencing of the IP_3_R1 and/or IP_3_R3 revealed a decrease in levels of cytosolic calcium in RCC4, A2780 and DLD1 cells (**c**). Interestingly, the increase in cytosolic calcium after AIK treatment was not as high as in cells treated with scrambled RNA (**c**). Double knockout of IP_3_R1/IP_3_R3 completely abolished apoptosis induction (**d**). Further, we compared apoptosis induction (**e**) in DLD1 and DLD1/IP_3_R3_del cells after 24 and 48 h hypoxia induced by DMOG. Depletion of the IP_3_R3 resulted in rapid increase of apoptosis both, in normoxia and hypoxia. On contrary to DLD1 cells, this increase was not dependent on duration of hypoxia (**e**) in DLD1/IP_3_R3_del cells, but the level of the apoptosis was higher in normoxic and 24 h hypoxia group compared to DLD1 cells. Scale bar represents 300 μm. Results are displayed as mean ± SEM, *n* = 3–6. Statistical significance *p < 0.05, **p < 0.001, and ***p < 0.0001

To evaluate mutual interaction of IP_3_R1 and IP_3_R3, we compared apoptosis induction in DLD1 and DLD1/IP_3_R3_del cells after silencing of the IP_3_R1 and subsequent induction of apoptosis by AIK (Fig. 4a). Silencing of the IP_3_R1 decreased the basal apoptosis compared to cells treated with scrRNA in both, DLD1 and DLD1/IP_3_R3_del cells. However, after the treatment with AIK apoptosis was significantly higher in DLD1/IP_3_R3_del than in DLD1 cells. In DLD1 cells we observed co-localization of IP_3_R1 and IP_3_R3 (Fig. 4b). Silencing of the IP_3_R1 and/or IP_3_R3 revealed a decrease in levels of cytosolic calcium in RCC4, A2780, and DLD1 cells (Fig. 4c). Interestingly, the increase in cytosolic calcium after AIK treatment was not as high as in cells treated with scrambled RNA (Fig. 4c). Double knockout of IP_3_R1/IP_3_R3 completely abolished apoptosis induction (Fig. 4d). Further, we compared apoptosis induction (Fig. 4e) in DLD1 and DLD1/IP_3_R3_del cells after 24 and 48 h hypoxia induced by DMOG. Depletion of the IP_3_R3 resulted in rapid increase of apoptosis both, in normoxia and hypoxia. On contrary to DLD1 cells, this increase was not dependent on duration of hypoxia (Fig. 4d) in DLD1/IP_3_R3_del cells, but the level of the apoptosis was higher in normoxic and 24 h hypoxia group compared to DLD1 cells.

**Fig. 5:**
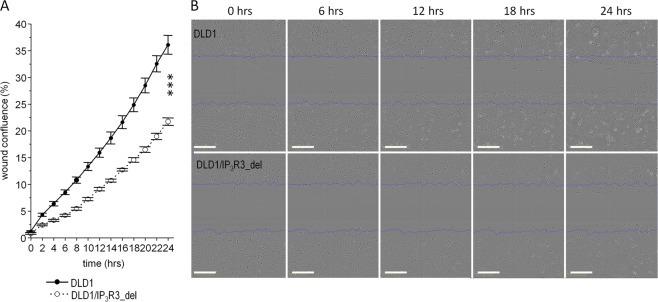
Migration of DLD1 and DLD1/IP_3_R3_del cells. In DLD1/IP_3_R3_del cells, inhibition of migration was time-dependent up to 24 h (**a**). Typical images immediately after a scratch and after 6, 12, 18, and 24 h are shown (**b**). Scale bar represents 300 μm. Results are displayed as mean ± SD, *n* = 8. Statistical significance after 14 h was evaluated by nonparametric Mann–Whitney *U* test, ***p < 0.0001

Also, we tested the effect of IP_3_R3 depletion on cell migration (Fig. 5). We observed a significant decrease in DLD1/IP_3_R3_del migration compared to DLD1 migration in a time-dependent manner up to 24 h.

## Discussion

In this work, we have clearly proved that in tumor cells of solid tumors, the type 3 IP_3_R has anti-apoptotic and proliferative function, on contrary to the type 1 and type 2 IP3 receptors, where pro-apoptotic effect was described.

Pro-apoptotic effect of the type 1 IP_3_R is well documented in several papers from our laboratory^5,6,21^, and also from others e.g., ref. ^22^. Also, pro-apoptotic effect was ascribed to the type 2 IP_3_R^7,8,23^. Nevertheless, despite some recent papers dealing with the function of the IP_3_R3^12,24^, the function of these receptors in tumor cells was not fully understood yet. Thus, we started with microarray profiling of samples from ccRCC and compared these samples to corresponding unaffected tissue from the same patient. We observed that the IP_3_R1 was decreased in 50% of samples and not changed in another 50%. The IP_3_R2 was also decreased in ccRCC compared to normal tissue from the same patient in 83.3% and not changed in 16.7%. The IP_3_R3 was increased in 77.8% ccRCC and not changed in 22.2%. These results clearly point to the different function of the IP_3_R3 compared to IP_3_R1 and IP_3_R2 in tumors—at least in ccRCC. Therefore, we studied the involvement of the IP_3_R1 and IP_3_R3 on stable cell lines derived from ccRCC—RCC4, but also on ovarian cancer cell line—A2780 and colorectal carcinoma cell line—DLD1. Because of the lack of specific blockers of these receptors, we silenced each receptor by the corresponding siRNA and determined the apoptosis after induction by AIK. As expected, silencing of the IP_3_R1 resulted in suppressing AIK-induced apoptosis, which is in line with the proapoptotic effect of this receptor described in the literature. The AIK-induced apoptosis after silencing of the IP_3_R3 was increased in all three cell lines—A2780, DLD1, and RCC4, which points to the anti-apoptotic effect of the IP_3_R3. These results nicely correspond with the expression profile observed on samples of ccRCC.

Further, we decided to study the functional consequences of the IP_3_R3 on tumor growth and migration. Using CRISPR/Cas9 method, we constructed DLD1 cell line without functional IP_3_R3 (DLD1/IP_3_R3_del). We injected the same amount of DLD1 and/or DLD1/IP_3_R3_del cells into nude mice and evaluated the tumor's growth. We observed that the volume of tumors from DLD1/IP_3_R3_del cells was significantly lower compared to DLD1 cells. Moreover, xenografts from DLD1/IP_3_R3_del cells were apoptotic, probably due to rapidly increased expression of the IP_3_R1. Migration of the DLD1/IP_3_R3_del cells was suppressed compared to DLD1 cells, which points to the positive effect of the IP_3_R3 on cell’s migration. Decreased migration due to blocking IP_3_R3 was observed also by Mound et al.^12^ in breast cancer cells. These authors used three human breast cancer cell lines with different migration velocities and they observed that a higher IP_3_R3 expression level, but not IP_3_R1 nor IP_3_R2, is correlated to a stronger cell line migration capacity and a sustained calcium signal. Silencing of the IP_3_R3 led to a significant decrease of cell migration capacities of all three breast cancer cell lines.

The question remains, what is the mechanism by which IP_3_R3 realized its anti-apoptotic effect. On contrary to IP_3_R3, the involvement of the IP_3_R1 in apoptosis induction in a variety of cells was shown in several papers^6,25^. The IP_3_R1 is involved in the mechanism of action of some potential chemotherapeutic agents, e.g., sulforaphane^6^, or melatonin^21^, partially through the increased expression of the IP_3_R1. Treatment of DLD1 cells with AIK resulted in increased expression of the IP_3_R1 and decreased expression of the IP_3_R3 in parallel. This observation would suggest that the ratio of the IP_3_R1/IP_3_R3 is responsible for pro- or anti-apoptotic response. By PLA, we detected heterocomplexes of the IP_3_R1/IP_3_R3 in DLD but not DLD1/IP_3_R3_del cells. Thus, we compared apoptosis before/after treatment with AIK in DLD1/IP_3_R3_del cells with silenced IP_3_R1 and also in cells with double knockouted IP_3_R1/IP_3_R3 with single silenced IP_3_R1 or single knockout DLD1/IP_3_R3_del. In the double knockouted cells DLD1/IP_3_R1/IP_3_R3_del and also in DLD1/IP_3_R3_del cells with silenced IP_3_R1, AIK induces an additional increase in apoptosis. These results would suggest that the anti-apoptotic mechanism of IP_3_R3 does not solely include upregulation of the IP_3_R1.

Hypoxia is one of the important characteristics of a majority of solid tumors, Moreover, the ccRCC tumors are spontaneously hypoxic, since Von Hippel-Lindau (VHL) is an important tumor suppressor that is lost in the majority of ccRCC. The loss of VHL leads to HIF accumulation and translocation into the nucleus, which subsequently activates the transcription of HIF target genes that are involved in critical oncogenic pathways^26^. Although our results with IP_3_R1 and IP_3_R3 silencing were the same in pseudohypoxic RCC4 cells and normoxic A2780 and DLD1 cells, we decided to compare the effect of hypoxia on apoptosis induction in DLD1 and DLD1/IP_3_R3_del cells. As expected, in control, normoxic DLD1/IP_3_R3_del cells, the percentage of Annexin V positive cells was approximately twice as high as in DLD1 cells. In DLD1 cell, the number of apoptotic cells increased, but in DLD1/IP_3_R3_del cells it remains on control levels. These results also point to the anti-apoptotic effect of the IP_3_R3 in tumor cells.

In summary, all our experiments, which were performed either on human samples or in vitro by silencing and apoptosis determination or in vivo on nude mice strongly suggest the anti-apoptotic role of the IP_3_R3. However, the mechanism of this action remains to be further elucidated.

## Supplementary information

Supplemental data
