# Supplementary material for: Type 3 inositol 1,4,5-trisphosphate receptor has antiapoptotic and proliferative role in cancer cells
Source: Cell Death Dis. 2019 Feb 22;10(3):186. doi: 10.1038/s41419-019-1433-4 (PMC6385365; doi:10.1038/s41419-019-1433-4)
Supplement: Supplementary file 1 — Supplemental data [file 41419_2019_1433_MOESM1_ESM.pptx]

## Slide 1
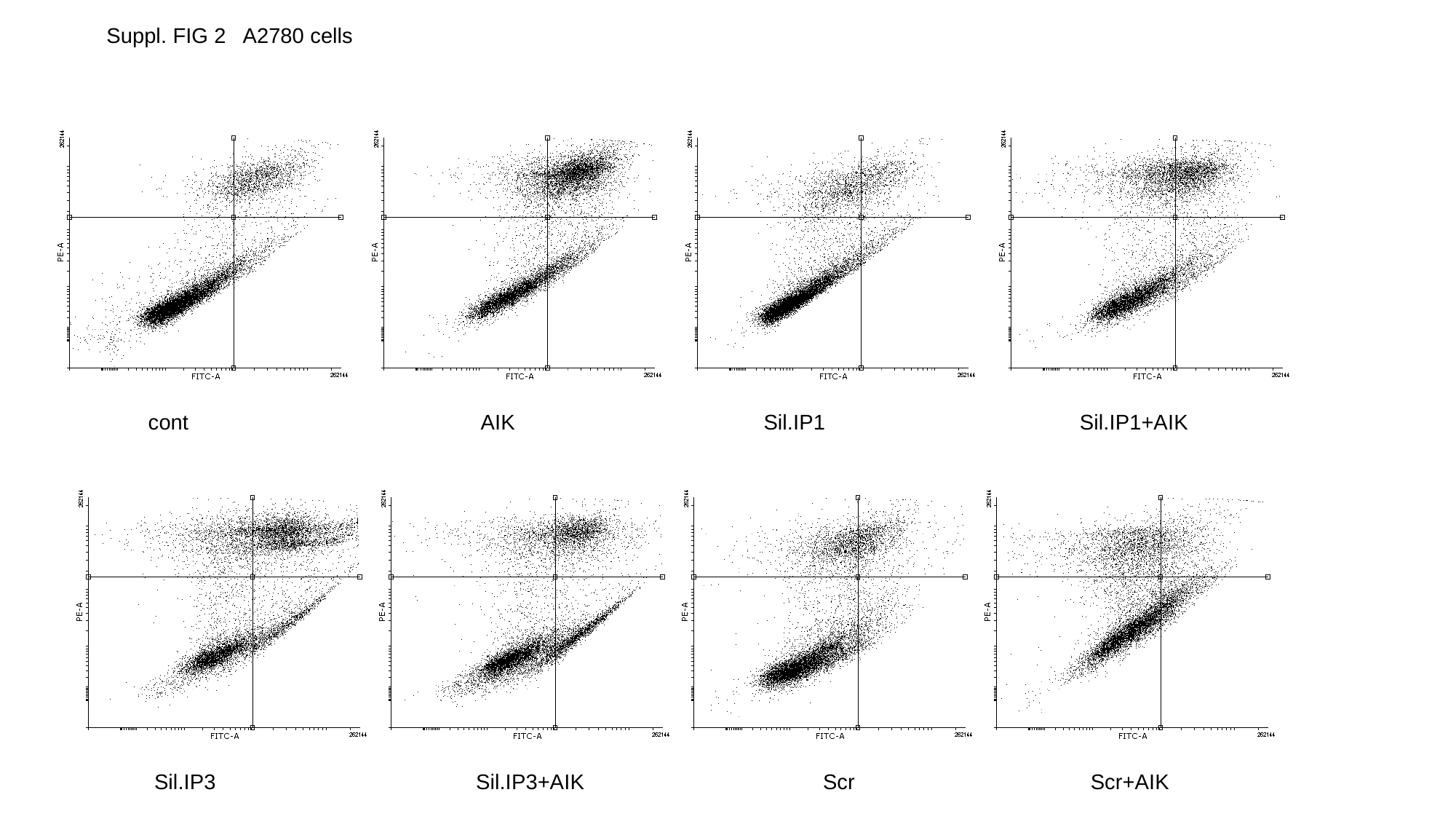

Suppl. FIG 2 A2780 cells
cont
AIK
Sil.IP1
Sil.IP1+AIK
 Sil.IP3
Sil.IP3+AIK
Scr
Scr+AIK

## Slide 2
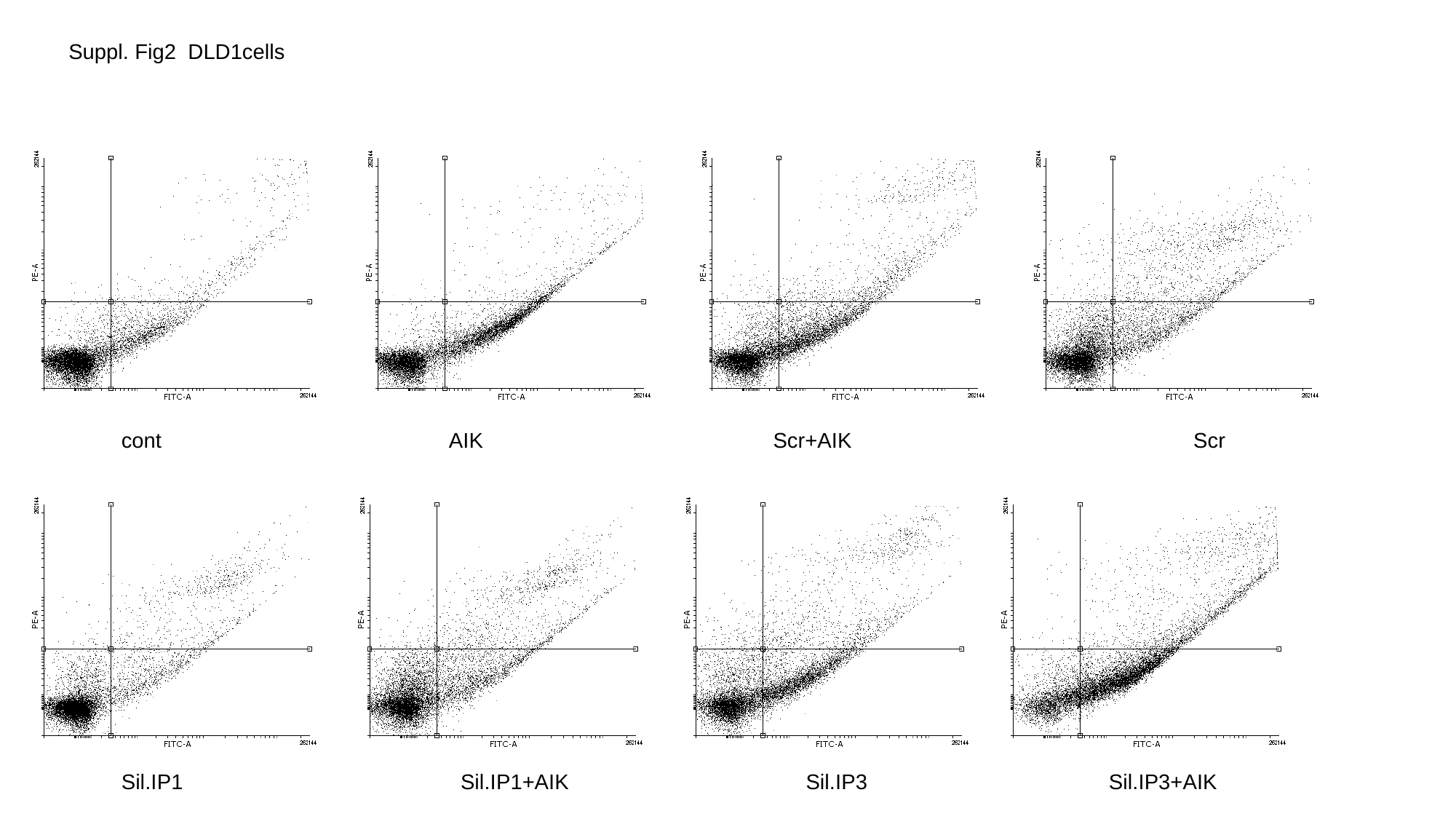

Suppl. Fig2 DLD1cells
cont
AIK
Scr+AIK
 Scr
Sil.IP1
Sil.IP1+AIK
Sil.IP3
Sil.IP3+AIK

## Slide 3
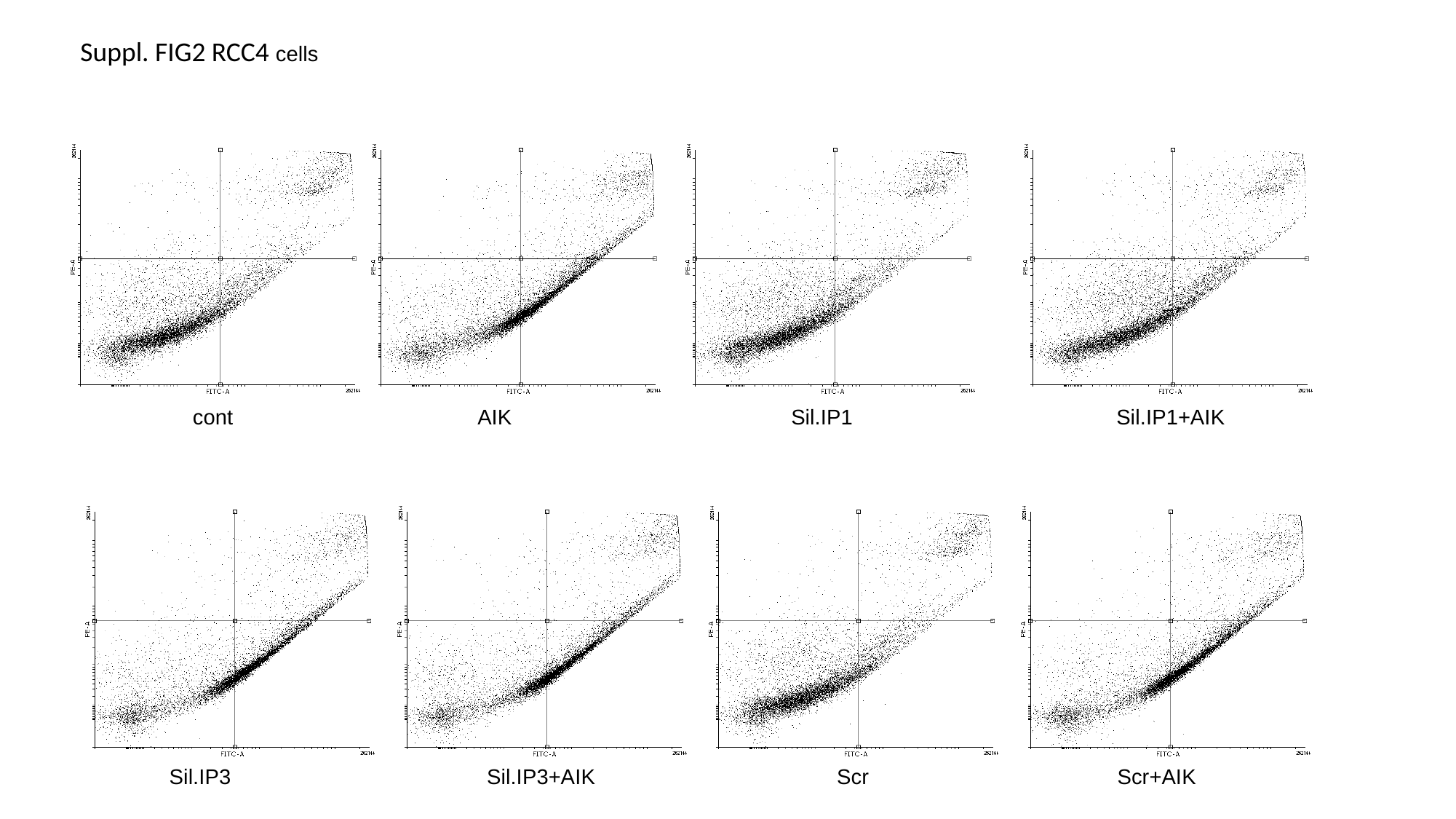

Suppl. FIG2 RCC4 cells
cont
AIK
Sil.IP1
Sil.IP1+AIK
Sil.IP3
Sil.IP3+AIK
Scr
Scr+AIK

## Slide 4
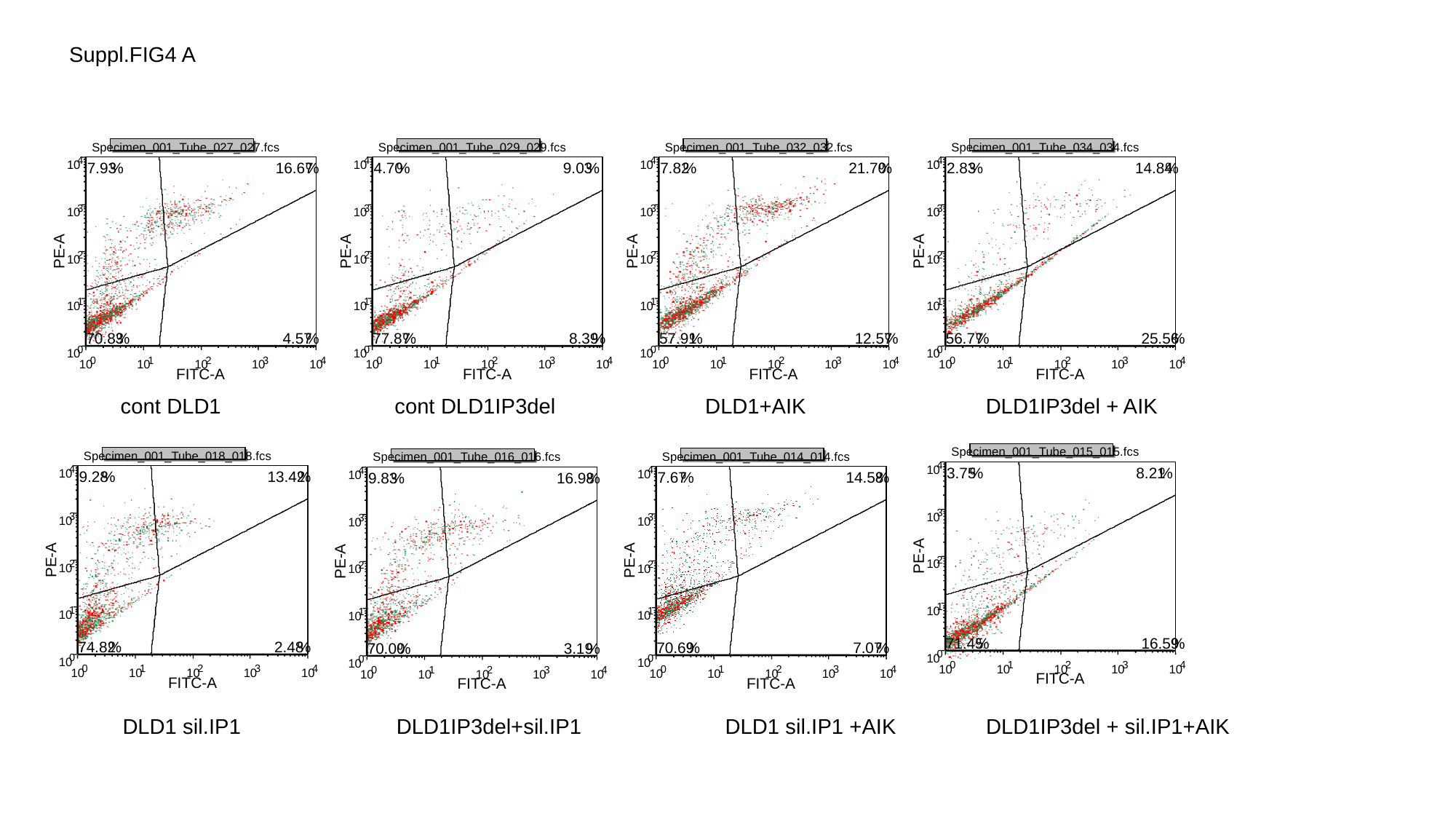

Suppl.FIG4 A
Specimen_001_Tube_027_027.fcs
4
10
7.93
%
16.67
%
3
10
PE-A
2
10
1
10
70.83
%
4.57
%
0
10
0
1
2
3
4
10
10
10
10
10
FITC-A
Specimen_001_Tube_029_029.fcs
4
10
4.70
%
9.03
%
3
10
PE-A
2
10
1
10
77.87
%
8.39
%
0
10
0
1
2
3
4
10
10
10
10
10
FITC-A
Specimen_001_Tube_032_032.fcs
4
10
7.82
%
21.70
%
3
10
PE-A
2
10
1
10
57.91
%
12.57
%
0
10
0
1
2
3
4
10
10
10
10
10
FITC-A
Specimen_001_Tube_034_034.fcs
4
10
2.83
%
14.84
%
3
10
PE-A
2
10
1
10
56.77
%
25.56
%
0
10
0
1
2
3
4
10
10
10
10
10
FITC-A
 cont DLD1 cont DLD1IP3del DLD1+AIK DLD1IP3del + AIK
Specimen_001_Tube_015_015.fcs
4
10
3.75
%
8.21
%
3
10
PE-A
2
10
1
10
71.45
%
16.59
%
0
10
0
1
2
3
4
10
10
10
10
10
FITC-A
Specimen_001_Tube_018_018.fcs
4
10
9.28
%
13.42
%
3
10
PE-A
2
10
1
10
74.82
%
2.48
%
0
10
0
1
2
3
4
10
10
10
10
10
FITC-A
Specimen_001_Tube_014_014.fcs
4
10
7.67
%
14.58
%
3
10
PE-A
2
10
1
10
70.69
%
7.07
%
0
10
0
1
2
3
4
10
10
10
10
10
FITC-A
Specimen_001_Tube_016_016.fcs
4
10
9.83
%
16.98
%
3
10
PE-A
2
10
1
10
70.00
%
3.19
%
0
10
0
1
2
3
4
10
10
10
10
10
FITC-A
DLD1 sil.IP1 DLD1IP3del+sil.IP1 DLD1 sil.IP1 +AIK DLD1IP3del + sil.IP1+AIK

## Slide 5
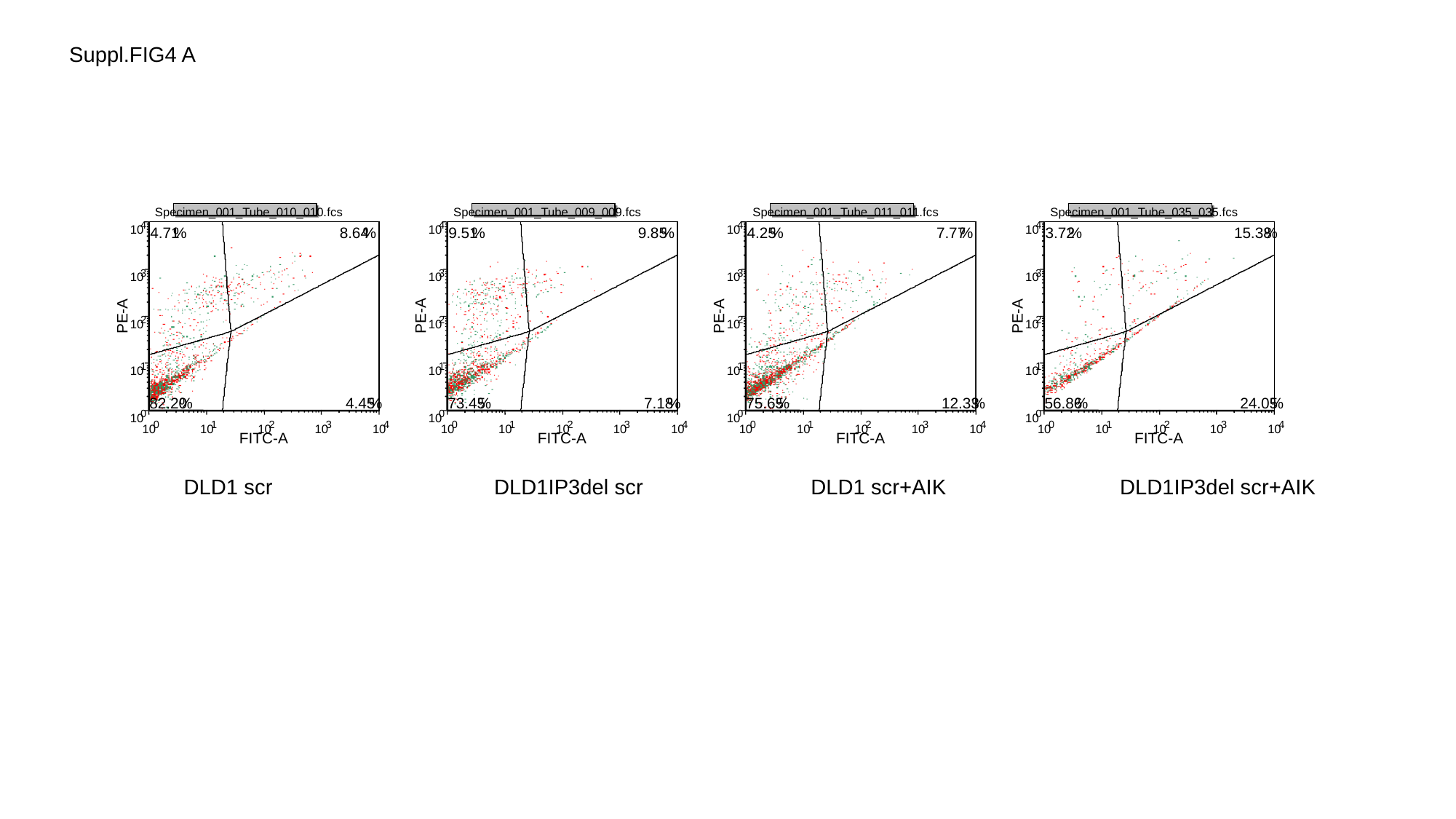

Suppl.FIG4 A
Specimen_001_Tube_010_010.fcs
4
10
4.71
%
8.64
%
3
10
PE-A
2
10
1
10
82.20
%
4.45
%
0
10
0
1
2
3
4
10
10
10
10
10
FITC-A
Specimen_001_Tube_009_009.fcs
4
10
9.51
%
9.85
%
3
10
PE-A
2
10
1
10
73.45
%
7.18
%
0
10
0
1
2
3
4
10
10
10
10
10
FITC-A
Specimen_001_Tube_011_011.fcs
4
10
4.25
%
7.77
%
3
10
PE-A
2
10
1
10
75.65
%
12.33
%
0
10
0
1
2
3
4
10
10
10
10
10
FITC-A
Specimen_001_Tube_035_035.fcs
4
10
3.72
%
15.38
%
3
10
PE-A
2
10
1
10
56.86
%
24.05
%
0
10
0
1
2
3
4
10
10
10
10
10
FITC-A
DLD1 scr DLD1IP3del scr DLD1 scr+AIK DLD1IP3del scr+AIK

## Slide 6
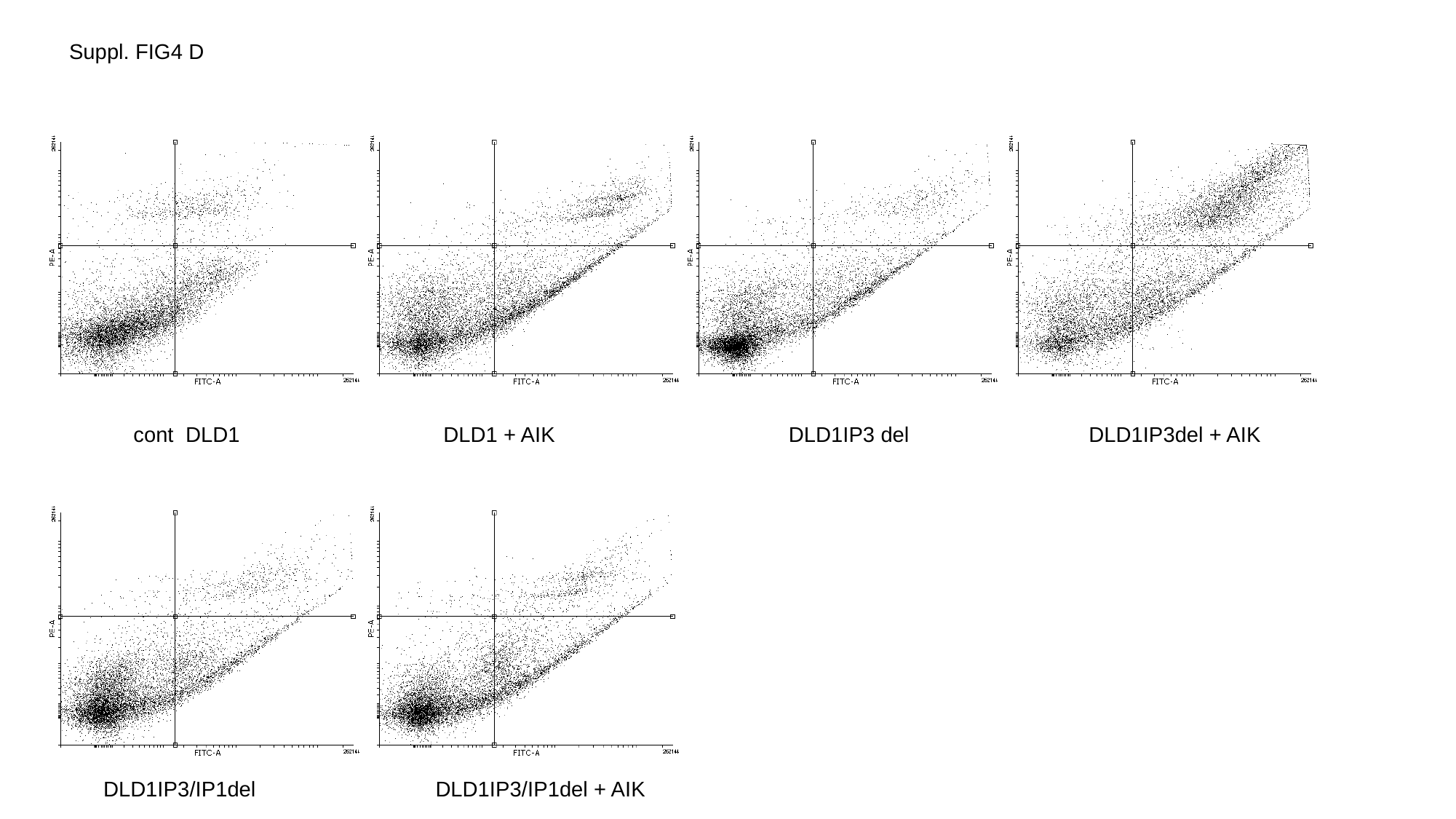

Suppl. FIG4 D
 cont DLD1 DLD1 + AIK DLD1IP3 del DLD1IP3del + AIK
DLD1IP3/IP1del DLD1IP3/IP1del + AIK
